# Phenotype Transformation of PitNETs

**DOI:** 10.3390/cancers16091731

**Published:** 2024-04-29

**Authors:** Zhenwei Li, Yinzi Wu, Guannan He, Renzhi Wang, Xinjie Bao

**Affiliations:** Department of Neurosurgery, Peking Union Medical College Hospital, Chinese Academy of Medical Sciences and Peking Union Medical College, Beijing 100050, China; drlzw990504@163.com (Z.L.); wuyinzi@email.szu.edu.cn (Y.W.); heguannandoc@163.com (G.H.); wangrz@126.com (R.W.)

**Keywords:** pituitary adenoma, pituitary neuroendocrine tumor, phenotype transformation, recurrence, silent pituitary adenoma

## Abstract

**Simple Summary:**

Pituitary neuroendocrine tumors are derived from the clonal expansion of a single anterior pituitary cell and are thought to retain their secretory patterns alongside their growth. However, cases of phenotype transformation in pituitary neuroendocrine tumors have been reported continuously. These tumors represented various transformation during the course and the phenomenon is rare and unexpected. We reviewed cases with phenotype transformation and defined the phenomenon. We also determined the clinical characteristics of these cases and the treatment of them. The possible mechanisms beneath this rare biological behavior were discussed in detail. Surgeons need diagnose and predict these tumors more accurately to reserve the precious specimens.

**Abstract:**

Phenotype transformation in pituitary neuroendocrine tumors is a little-known and unpredictable clinical phenomenon. Previous studies have not clearly defined and systematically concluded on the causes of this rare phenomenon. Additionally, the mechanisms of phenotype transformation are not well known. We reviewed cases reported in the literature with the aim of defining phenotype transformation in pituitary neuroendocrine tumors. We present an overview of the wide spectrum of phenotype transformation and its clinical features. We also discuss findings on the potential mechanism of this rare transformation, which may be related to PC1/3, the bioactivity of secretory hormones, gene mutations and the plasticity of pituitary neuroendocrine tumors. Clinicians should be aware of this rare phenomenon and more studies on the underlying mechanisms are required.

## 1. Introduction

Pituitary neuroendocrine tumors (PitNETs) are neoplasms that originate from neuroendocrine cells in the adenohypophysis [1]. They are the second most common intracranial tumors, accounting for 15–20% of them [1]. For most PitNETs, trans-sphenoidal surgical resection is the first-line treatment option. In the case of non-feasibility or failure of surgery because of invasive/recurrent lesions, multimodal therapy is considered, including temozolomide, dopamine agonists, somatostatin analogs, other medical therapy, radiation therapy, and even palliative bilateral adrenalectomy [2,3,4]. More therapies, including immunotherapeutic strategies to affect the tumor micro-environment for instance, are also being developed [5]. The various types of PitNETs are determined by the hypersecreted hormones and clinical features of the tumors. Somatotroph tumors secrete growth hormone and cause acromegaly, lactotroph tumors secrete prolactin and induce hyperprolactinemia, thyrotroph tumors produce excess thyroid stimulating hormone (TSH) and lead to mild central hyperthyroidism, corticotroph tumors secrete adrenocorticotrophic hormone and cause Cushing’s disease (CD) and a small number of gonadotroph tumors secrete intact follicle-stimulating hormone (FSH) or luteinizing hormone (LH) and may cause sexual dysfunction and hypogonadism. Some types of PitNETs even express multiple hormones [6]. Additionally, some PitNETs, called silent PitNETs (22% to 54% in different series), express pituitary hormones or their transcription factors measured with immunohistochemistry (IHC) but without clinical symptoms [7]. The 5th Edition of the WHO Classification of Endocrine and Neuroendocrine Tumors (2022) proposes that PitNETs be classified on the basis of hormones, transcription factors and other biomarkers, as determined by IHC findings [6].

Previous studies indicate that most PitNETs are initiated by the expansion of a single cell [8] and they maintain the same secretory pattern during the whole course of disease, unlike some malignancies that exhibit heterogeneity [9]. However, some silent corticotroph tumors were observed to change to CD [10], indicating a switch between different phenotypes. The phenotype switching of PitNETs has also gradually been reported in other types of PitNETs. This phenotype transformation is relatively rare and lacks a widely agreed name and definition; furthermore, the underlying mechanisms remain unknown. Here, we review PitNETs that exhibit phenotype transformation and discuss the possible mechanisms.

## 2. Definition

**Figure 1:**
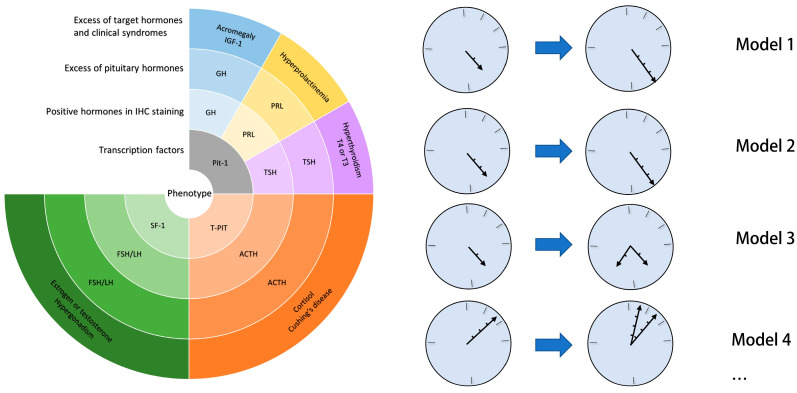
PitNET phenotype clock. The pointers denote the hormone secretory model of the PitNET in a phase. IHC, immunochemistry; GH: growth hormone; PRL: prolactin; Pit-1: pituitary specific transcription factor 1; SF-1: steroidogenic factor 1 T-PIT: T-box family member TBX19; CD: Cushing’s disease.

The transformation of phenotype in PitNETs is rarely reported and the definition is vague. Terms such as “transformation”, “changing phenotype”, “metamorphosis” and “modification” have been used to describe this rare biological behavior [7,11,12,13,14,15]. The diagnosis of PitNETs is based on the IHC staining of hormones and transcription factors, the pattern of secretary pituitary hormones and clinical symptoms [6]. Broadly speaking, any change in the four dimensions between two phases can be called a transformation of phenotype in PitNETs (Figure 1) [13,15,16,17]. 

As shown in Figure 1, we can describe the state of a PitNET using the pointers on a “clock”. With a simple clock face, we can represent the expression condition of a PitNET. The transformation is represented by the difference between the length, direction and number of pointers. The depth of color on the clock face represents the expression condition of each hormone. We used gradually deepening colors to represent progressively functional hormone expression (from totally silent to functional) [18]. In other words the length of a pointer stands for whether the hormones are silent or functional at the current stage. For example, a short pointer represents one type of silent PitNETs, a long pointer represents a functional PitNET with clinical syndromes and multiple pointers denote a PitNET that secretes multiple hormones. We have listed some types of transformations reported (models 1–4), and will discuss their possible mechanisms. Model 1 is the most common transformation type, where an SCA [IHC-ACTH(+), serum ACTH(−)] transforms to CD; model 2 represents a clinically SCA [serum ACTH(+) without CD symptoms] transformed to CD. Models 3 and 4 are both associated with the co-expression of multiple hormones. Model 3 denotes the transformation of SCA to PitNET with the co-expression of ACTH and FSH/LH measured by IHC but without clinical syndromes; model 4 means a somatotroph tumor switch to a mammosomatotroph tumor. Of note, there are many cases which cannot be classified using these models due to their wide spectrums of phenotype transformation.

## 3. Literature Review 

**Figure 2:**
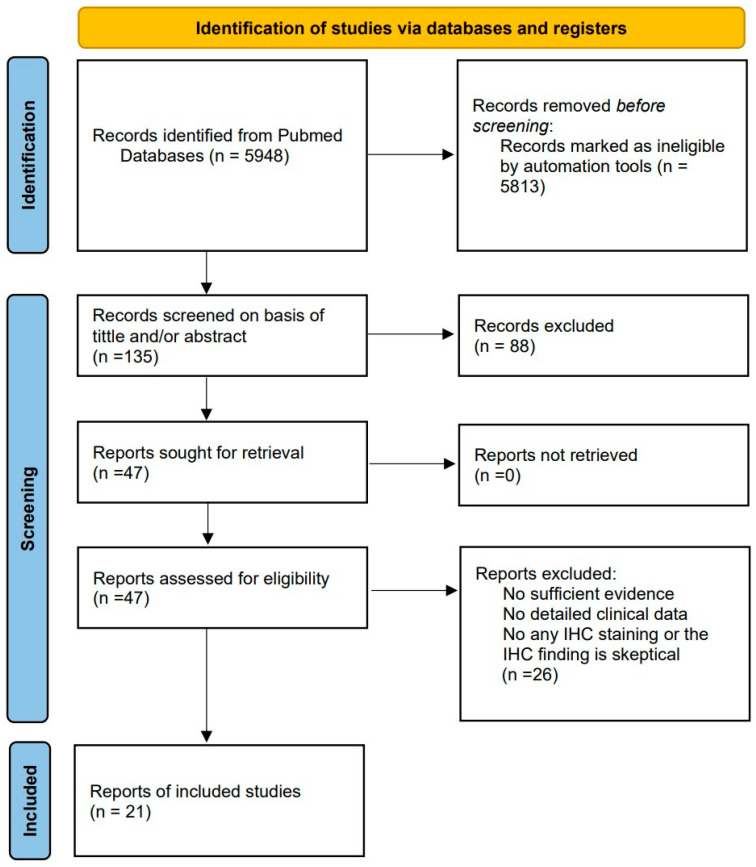
Search and selection strategy of case reports for this review.

We searched published case reports on PitNETs that exhibited phenotype transformation using the PubMed database. The keywords included ([PitNETs] OR [pituitary adenoma]) AND ([change] OR [transformation] OR [metamorphosis] OR [reversal]) (Figure 2). Notably, some cases may have been missed in the literature search because there is no clear term to define the transformation. We included case reports published in English describing human PitNET phenotype transformation using the keywords listed above. The exclusion criteria were as follows: (1) there was no clear shift in PitNET phenotype; (2) the report did not include at least one IHC staining result for evidence of the phenotype shift; (3) detailed clinical data were not described; and (4) reports were published in languages other than English. Finally, we included 27 cases from 21 articles that met the inclusion/exclusion criteria.

## 4. Clinical Characteristic

The prevalence of the rare transformation was not well-estimated due to the absence of a clear definition and large retrospective reviews. An operation series reviewed 1023 patients with PitNETs from a single center; 65 patients (6.4%) exhibited recurrence and 5 (7.7%) had tumors that changed phenotype [13]. The clinical data of the 27 included patients are summarized in Table 1 [7,9,10,11,12,13,15,16,19,20,21,22,23,24,25,26,27,28,29,30,31]. The 27 patients included 8 males and 19 females (M:F, 1:2.38). The average age at first diagnosis of PitNET was 43.5 ± 14.2 years, ranging from 17 to 77 years. The interval from the initial diagnosis of PitNETs to the transformation ranged from 1 to 20 years, with an average of 6.25 ± 4.19 years. The transformation often occurred following recurrence of the tumor after surgery. However, in three cases, phenotype switching emerged during surveillance or medicine treatment (cases 12, 15 and 24). The treatments or events before the transformation were surgery (88.9%), radiotherapy (33.3%) or pituitary apoplexies with clinical symptoms (7.4%), which might promote the transformation of PitNETs as in some malignant tumors [13]. 

The spectrum of the transformation of PitNET phenotype is wide. The transformation of clinical syndromes can be classified into three types: functionalization of silent PitNETs (19/26), silence of functional PitNETs (4/26) and switch between different lineages (3/26). The clinical syndromes of patient 28 were not detailed. Among the 19 patients with functionalization of silent PitNETs, 15 tumors transformed to CD (one transformed to pituitary carcinoma); this was the most common phenotype transformation. Of the five patients that initially had a functional PitNET, four cases were ACTH-secreting PitNETs that changed to NFP. Cases with different secretory hormones in two clinical phases were rare: two patients with hyperprolactinemia developed acromegaly and one patient with a silent gonadotroph tumor developed CD. Additionally, nine cases had a phase in which hormones from more than one lineage were co-expressed (model 3 in Figure 1). This phenomenon was mainly observed in PitNETs from the Pit-1 lineage (4/6, 66.6%), and this was also observed in ACTH-secreting tumors. Some studies proposed that the co-expression of other hormones may be from nontumorous cells in the lesion [28]. Data on transcription factors were only reported for case 7.

Information on the treatment and outcome of these patients is also summarized in Table 1. Among the cases with detailed follow-up data, the average follow-up was 4.80 ± 2.98 years. The treatment and outcome of these tumors varied. Multiple types of treatments, including medicine, radiotherapy and surgery, were used for tumor removal and relief of clinical symptoms. Approximately 74.1% patients (20/27) underwent at least one tumor removal surgery and 5 underwent multiple surgeries. Seven patients (25.9%) exhibited tumor recurrence after surgery after phenotype transformation, indicating these cases might be classified as refractory pituitary adenomas. Radiotherapy was considered in 14 patients, in most cases in combination with surgery. Four patients who did not undergo surgery achieved biological remission or stable even shrunk PitNETs after radiotherapy. However, in eight challenging cases, the tumors were out of control after the earlier mentioned treatment. Notably, six cases were severe CD after transformation and four received bilateral adrenalectomy to control severe CD. The PitNET of case 14 recurrent in a short term and resisted multiple treatment, including TMZ. Seven patients presented with hypoadrenalism and required pituitary hormone replacement after surgery; notably, all of the tumors were silent cortitroph adenomas (SCAs) before transformation. A high frequency of post-operative hypopituitarism (35%) in SCA was previously reported [32]. Two patients died from complications of tumor and surgeries; the cause of death was not detailed. For these patients, physicians should maintain a long-term follow-up and multiple therapies in the early stage. 

**Table 1:** The phenotype transformation of the included patients.

Case Number	Authors and Reference	Age/Sex	Events before Transformation	Time up to Transformation (Years)	IHC Staining before the Transformation	IHC after the Transformation	Clinical Diagnosis before Transformation	Clinical Diagnosis after Transformation	Treatment after Transformation	Follow-Up after Transformation (Years)	Outcome and Adverse Events
1	Demarchi, 2022 [19]	65/F	Surgery, apoplexy	6	ACTH (+)	ACTH (−) OR (strongly +)	NFP	CD	Surgery, RT, TMZ, ketoconazole, cabergolin	7	RR, BR
2	Brown, 2020 [16]	56/M	Multiple surgeries, apoplexies	11	1st: FSH/LH (weakly +), 2nd: all hormones (±)	3rd, 4th: ACTH (+)	NFP	NFP	Multiple surgeries, RT	2	Multiple recurrences, lost to follow-up apoplexies, hypopituitarism
3	Guerrero-Pérez, 2020 [9]	24/F	Surgery	1.5	ACTH (strongly +)	NA	CD	NFP	RT	4	PR
4		77/F	Surgery	5	GH strongly (+), PRL focally (+)	NA	Acromegaly	NFP	RT	3	PR, TSH deficiency
5	Rotman, 2019 [22]	51/M	Surgery, radiotherapy	14	NA	ACTH (strongly +)	CD	Silent corticotroph pituitary carcinoma	Surgery, RT, TMZ, Bevacizumab	8	Transformation to pituitary carcinoma, SD
6	Jahan, 2019 [21]	18/M	Surgery	3	NA	ACTH/FSH (+)	NFP	CD	Surgery, RT	NR	NR
7	Andino-Ríos, 2018 [20]	41/F	Surgery	3	FSH/ LH /SF-1 (+)	FSH (strongly +) LH (+)	NFP	Excess of FSH Hypergonadism	Surgery	NR	NR
8	Zoli, 2015 [15]	47/F	Multiple surgeries	7	ACTH (10% +)	ACTH (80% +)	NFP	CD	Surgery, multiple RT, bilateral adrenalectomy	7	RR, BR
9		51/M	Multiple surgeries	1	ACTH (90% +)	ACTH (80% +)	CD	NFP	Multiple surgeries	11	Recurrence, SD hypoadrenalism
10	Zoli, 2015 [15]	18/F	Surgery	6	ACTH (20% +)	ACTH (70% +)	NFP	CD	Surgery	2	RR, BR hypocorticism
11		37/F	Surgery	2	ACTH (90% +)	ACTH (75% +)	Excess ACTH but normal serum cortisol	CD with an extremely high ACTH concentration	Multiple surgeries	6	Multiple recurrences, hypoadrenalism, excess of ACTH
12		48/F	Clinical surveillance	2	NA	ACTH (80% +)	NFP	CD	Surgery	5	RR, transient hypocorticism
13	Lee, 2014 [23]	53/F	Multiple surgeries, radiotherapy	6	ACTH (+)	NA	NFP	CD	RT, mitotane and cabergoline	0.25	SD, BR
14	Batisse, 2013 [33]	47/M	surgery, radiotherapy	9	GH (5% +)	GH (40% +)	NFP	Acromegaly	Surgery, cisplatin, adriblastin, TMZ	3	Recurrence of a giant adenoma, SD, active acromegaly, bilateral blindness
15	Dessimoz, 2011 [17]	32/F	Dopamine agonist	10	NA	GH, PRL (strongly +)	Hyperprolactinemia	Hyperprolactinemia Acromegaly	Surgery	1	RR, BR
16	Lania, 2010 [24]	31/F	Dopamine agonist, multiple surgeries, multiple radio	13	PRL (+)	PRL (+) GH (10% +)	Hyperprolactinemia	Hyperprolactinemia Acromegaly	Surgeries, RT, lanreotide	3.5	Recurrence, hyperprolactinemia, bilateral blindness, death due to complications
17	Daems, 2009 [7]	41/M	surgery	7	GH (strongly +), PRL/TSH/α-subunit (diffused +)	NA	NFP	Acromegaly	Octreotide, pegvisomant	11	PR, BR
18	Daems, 2009 [7]	40/M	surgery	14	α-subunit (10% +), TSH (2–3% +)	α-subunit (25% +), TSH (20% +)	NFP	Hyperthyroid	surgery	NR	hypothyroid
19	Brown, 2007 [25]	32/F	Multiple surgeries, multiple radio	10	All hormones (−)	ACTH (− or strongly +)	NFP	Corticotroph pituitary carcinoma	Multiple surgeries	7	Recurrence, transformation to pituitary carcinoma, death
20	Salgado, 2006 [26]	49/F	Surgery, radiotherapy, bromocriptine	2	All hormones (−)	ACTH (>50% +) PRL/GH (slightly +)	NFP	CD	RT, ketoconazole	NR	NR
21	Sano, 2002 [28]	49/F	Multiple surgeries	1	ACTH (focally +)	ACTH (strongly +)	NFP	CD	Surgery, RT	NR	CD was not controlled
22	Kho, 2002 [27]	48/F	Multiple surgeries, radiotherapy	2	1st: LH/FSH (+) 2nd: all hormones (−)	NA	NFP	CD	Bilateral adrenalectomy	5	SD, CD was not controlled until adrenalectomy
23	Tan, 2000 [11]	39/F	Multiple surgeries	6	1,2: all hormones (−)	ACTH/PRL/FSH/GH (+)	NFP	CD	Surgery, RT, ketoconazole, bilateral adrenalectomy	4	CD was not controlled until adrenalectomy
24	Gheri, 1997 [12]	47/F	Clinical surveillance	3	NA	ACTH (+)	NFP with mild hyperprolactinemia	CD	Surgery, RT, ketoconazole, octreotide	NR	NR
25	Felix, 1991 [31]	19/M	Multiple surgeries	NA	ACTH (+)	2nd, 3rd, 4th: ACTH/FSH/LH/α-subunit (+)	NFP	Not reported	multiple surgeries	7	Multiple recurrence
26	Cooper, 1987 [30]	60/F	Surgery	5	NA	ACTH (+)	NFP	CD	RT, aminoglutethimide and metapyrone	3	BR, hypocorticism
27	Vaughan,1985 [10]	55/F	Multiple surgeries	13	all cell ACTH (+)	ACTH (partly +)	NFP	CD	surgery, chemotherapy, bilateral adrenalectomy	1	SD, CD was controlled until adrenalectomy

NFP: non-functional PitNETs; NA: not applicable; FSH: follicle-stimulating hormone; LH: luteinizing hormone; ACTH: adrenocorticotropic hormone; GH: growth hormone; PRL: prolactin; TSH: thyroid stimulating hormone; Pit-1: pituitary specific transcription factor 1; SF-1: steroidogenic factor 1; CD: Cushing’s disease; RT: radiotherapy; NR: not reported; RR: radical removal; PR: partial response; SD: stable disease; BR: biochemical remission; TMZ: temozolomide.

## 5. Is It Really Phenotype Transformation?

The transformation of the PitNET phenotype is a rare clinical phenomenon, especially in tumors other than SCAs. This raises the question of whether the functional PitNET after recurrence is the resected tumor. Some reports indicated that the tumor with the transformed phenotype was an independent new PitNET or a tiny and hidden double pituitary adenoma [34]. Although most transformations in PitNETs occur from residual tumors after surgery [15], some PitNETs produce these transformations just in clinical surveillance, and no double PitNETs were reported in these tumors [12,15,17]. Some tumors with a transformed phenotype were found to be monoclonal [24]. This evidence supports the existence of phenotype transformation in PitNETs, although these events are rare. In contrast, the transdifferentiation theory considers that the mixed PitNETs, also called “double pituitary adenomas,” may be derived from the phenotype transformation of one PitNET. In other words, cells of an already formed pituitary adenoma could transdifferentiate into another cell type and constitute a PitNET with different biological behaviors. However, only one of three double pituitary cases expressed the same transcription factors, indicating the two components might derive from a single PitNET lineage. Further studies are needed to support the transdifferentiation theory [34,35].

Some tumors only exhibited IHC staining change in two phases [13,16]. Some cases also showed co-expressed focal staining of multiple hormones. Therefore, these transformations may be attributed to the false positivity of IHC staining, especially in the early stage. Most of the included cases did not report transcription factor expression because of time limitations. The lack of information on transcription factor expression may also increase the risk of misdiagnosis. Therefore, the detection of transcription factors is necessary.

There is also a series of cases in which symptoms caused by functional PitNETs lead to biochemical remission after pituitary apoplexy, especially in acromegaly and CD. These biological events have been described as “spontaneous remission” or “silent reversal” of acromegaly or CD [36,37,38,39,40]. The remission is similar to the silent transformation of functional PitNETs, especially when the apoplexy lacks the typical symptoms. However, it is generally agreed that these events are attributed to the destruction of the tumor by the hemorrhagic infarction. Three possible reasons are as follows: First, while in some cases the reduction of hormones is selective for GH or ACTH, most remission is combined with hypopituitarism. Second, the pathologic finding of PitNETs after apoplexy also exhibits strongly positive IHC staining and does not correspond to the typical silent PitNET. Finally, some cases showed recurrence of acromegaly after remission. Therefore, we do not believe remission after apoplexy is equal to the silent transformation of PitNETs, unless IHC evidence is provided. 

## 6. Potential Mechanisms of Transformation

### 6.1. The Transformation from SCA to Cushing’s Disease

The functionalization of SCAs is the most common transformation of PitNETs (model 1 and 2, Figure 1), accounting for more than half of this case series, and the further study on this rare behavior also focused on the transformation of SCAs. Therefore, a separate discussion of this type is necessary. As is reported, “SCA” may not mean the complete absence of secretory ACTH in serum. Higher evidence of acquired postoperative in patients with SCA was reported compared with NFPA [41]. One explanation is that the SCAs are functional and depress the ACTH secreted by normal pituitary cells [32]. This might mean the transformation process is continuous and the evidence of transformation is underestimated. Why the SCAs evolve to CD remains unclear, and three possibilities have been hypothesized. 

#### 6.1.1. PC1/3 

The most attractive and persuasive theory for the mechanism of SCA is from studies on prohormone convertase 1/3 (PC1/3), one of the proopiomelanocortin (POMC) processing enzymes. PC1/3 cleaves POMC, the precursor of ACTH. The absence of PC1/3 may result in the accumulation and secretion of POMC or other ACTH precursors, which show low biological activity [42]. PC1/3 mRNA level is significantly lower in the SCAs than the CD cells and even lower than other non-functional PitNETs [43,44]. Additionally, changes in PC1/3 might explain why the SCA switches to CD. To date, only one study has focused on the transformation of SCAs [45]. In this study, PC1/3 mRNA and protein were analyzed by qRT-PCR and IHC staining in tissue specimens from both phases in three of the patients with tumor transformation from SCA to CD (model 1 in Figure 1). While PC1/3 expression was negative or weak in the three patients in the initial phase of SCA, strong expression was detected in recurrence with CD. The difference between the expression of PC1/3 in the two phases may explain the potential mechanism of the transformation. Why or how PC1/3 changed in the transformation remains unclear. One hypothesis is the transformation is from the accumulation of PC1/3 in the cells. It might be a continuous process spanning a long time before emergence of CD. The concept of “subclinical SCA” mentioned above may support this hypothesis. Holck et al. found that NFPA contains smaller secretory granules than functional tumors and suggested that with sufficient tumor bulk, any defect in packaging or exocytosis of ACTH may be overcome and thus lead to peripheral release of ACTH [46]. However, this cannot explain the transformation from CD-related PitNETs to SCAs. Alternatively, the expression of PC1/3 may be up-regulated for some reason, which is supported by the change in PC1/3 mRNA in the two phases. The mechanism of change in PC1/3 should be studied further.

#### 6.1.2. High-Molecular-Weight ACTH

Another theory about why SCA is silent is that SCAs secrete predominantly high-molecular-weight (HMW) ACTH, which lacks bioactivity and may compete with normal ACTH (1 to 39 amino acids, ACTH 1–39) at the receptor level [47]. As described in a case report, whether HMW ACTH can be tested by the ACTH kit depends on the clinical ACTH kit and the structure of HMW ACTH [32,48,49]. HMW ACTH always results in “clinically” SCAs, which are characterized by a high level of ACTH but normal serum cortisol levels and the absence of CD symptoms (model 2 in Figure 1) [48]. Notably, both CD tumors and SCAs produce HMW ACTH, and the “clinically” SCA produces higher amounts [47]. A high amount of the biologically inactive HMW ACTH may compete with ACTH 1-39 for the ACTH receptor, therefore hiding the effect of excessive ACTH 1–39. However, the binding capacities of HMW ACTH and ACTH 1-39 have not been compared. There is only one report of functionalization in clinically SCA. In the study by Zoli et al., a patient (case 11) exhibited an excess of ACTH during the entire disease course. She did not manifest Cushing’s disease until the ACTH concentration reached 1500 pg/mL (reference range 9–52 pg/mL) when the tumor recurred [15]. This may indicate that the functionalization of clinically SCA eventually occurs when the total ACTH level is high enough. How HMW ACTH is produced in PitNETs is not completely clear. The process of conversion from POMC to ACTH 1–39 involves two steps: cleavage and maturation. PC1/3 and 2 participate in the cleavage of POMC. In ectopic ACTH–producing tumors that produce HMW ACTH, the expression of PC1/3 mRNA is much lower than levels in NFPA and CD-related PitNETs. Moreover, ectopic ACTH–producing tumors produce ACTH 1–39 after transfection of the PC1/3 gene. Therefore, it is reasonable to speculate the low expression of PC1/3 results in the impaired cleavage procession [50]. However, the relationship of PC1/3 and HMW ACTH in SCA has not been quantitatively studied. In SCAs that produce HMW ACTH, PC1/3 and PC2 were IHC-positive, while chromogranin A, which functions in the maturation of secretory granules, was negative. This may indicate that a defective maturation process leads to HMM ACTH [48]. More study is required to determine the expression of PC1/3 in HWM ACTH-secreted PitNETs and CD-related PitNETs. 

#### 6.1.3. Multiple Hormone Tumors?

The co-expression of other hormones with ACTH in SCA is rare because the tumor is derived from the T-PIT lineage [31]. Notably, in some cases of functionalization in SCA, multiple hormones in the cells were observed (case 5, 12 and 14). Although Tan et al. proposed that these hormones were expressed by normal pituitary cells wrapped in the tumors [11,13,21,51], the tumors should have been diagnosed as multiple hormone tumors in these cases. The hypothesis about these PitNETs will be presented later.

### 6.2. The Plasticity of Multiple Hormone Tumors

Most PitNETs are derived from clonal expansion of a single anterior pituitary cell and they retain their secretory pattern alongside their growth, which was demonstrated by an X chromosome inactivation study [52]. However, this theory was challenged for a long time. The plasticity of the normal pituitary has been demonstrated by multiple studies. For example, studies have reported a variable decrease in pituitary GH concentrations in the second half of pregnancy, and somatotrophs are recruited to switch from GH to PRL production [53,54]. Additionally, PitNETs originate from and are classified from the various cell types. Therefore, some studies explored the plasticity of PitNETs. Xu et al. obtained pituitary adenoma stem-like cells from two pituitary adenomas (one a GH-secreting tumor, the other a NFPT). These cells formed neurospheres in vitro and generated daughter cells, which showed the capacity to differentiate into three neural lineages under the induction of GHRH, GnRH, PRL-releasing peptide and TRH. The studies proved the plasticity of PitNETs in vitro and may shed light on the transformation of these tumors [55,56,57].

Similar findings were also observed in vivo. Kovacs et al. described a case of acromegaly in which three groups of cells were observed: one secreted GH, another secreted ACTH and the third group secreted both. Immunoelectron microscopy conclusively demonstrated ACTH in the secretory granules of several somatotrophs. The authors speculated the GH-secreting cells transformed into neoplastic ACTH-secreting cells [29].

In the cases of transformation of PitNET phenotype, the proportion of tumors co-expressing other hormones (model 3 and 4 in Figure 1) is unusually high, especially in the Pit-1 lineages (4/6 cases) [7,9,13]. In the studies, the tumors were diagnosed as monohormonal tumors and co-expression was dismissed. From these findings, one hypothesis is possible: these tumors should have been diagnosed as plurihormonal tumors, which are rarely diagnosed and partly or completely silent [6,35]. Some plurihormonal tumors show variable positivity for one or more hormones [58]. Under these conditions, the clinical symptoms, which are often undetectable, can be diverse. For example, the immature PIT1-lineage tumor, previously known as silent subtype 3 adenoma, expresses no hormone or one/multiple pituitary hormones, and approximately 29% of these tumors can exhibit significant hormonal excess [13,59]. Another example is the silent corticogonadotroph adenoma proposed by Cooper et al., which might account for a proportion of SCA; these tumors originate from the common progenitor cell of corticotroph and gonadotroph pituitary cells [60]. These so-called SCA can co-express SF-1 and LH, like in case 2. Additionally, diagnosis of these tumors depends on transcription factor staining and electron microscopy, which can result in misdiagnosis. These immature plurihormonal tumors lack the features of terminally differentiated mature cell phenotypes [61]. They may be more primitive than monohormonal tumors [62]. It can be speculated these multiple hormone tumors are more plastic than terminally differentiated PitNETs and retain the capacity to differentiate or even reverse differentiate into PitNETs of other lineages. For example, cases 15 and 16 may essentially be mammosomatotroph tumors or acidophil stem cell tumors that co-express GH and PRL and these are considered immature in Pit-1 lineage tumors; the two clinical phases are different secretory pattern of these tumors. Future studies could explore this issue by focusing on the biological behavior of plurihormonal tumors. The clinical application of transcription factor staining and electron microscopy may help identify plurihormonal tumors.

### 6.3. Gene Mutation

Lania et al. found a mutation in the GNAS gene (present in approximately 40% of GH tumors) in the tissue of a patient (case 16) after the PRL-secreting PitNETs secreted GH and caused acromegaly (model 4 in Figure 1) that was absent in the previous surgical specimen. The authors also proved the monoclonality of the tumor through X-inactivation analysis, which excluded the possibility of the double PitNETs [24,63]. Thus, mutation of some oncogenes may be the underlying mechanism of this transformation. More genetic evidence is needed to prove this hypothesis. 

## 7. Conclusions and Prospect

Here, we presented a review of the published cases of PitNETs with phenotype transformation. We summarized the feature of the phenomenon and propose a specific definition of it. It is an unexpected and rare clinical phenomenon and has always been known that SCA causes CD when recurrent. In fact, PitNETs can undergo transformation of various aspects, including expression of hormones and transcription, secreted hormones and the corresponding syndromes. The tumors exhibit a wide spectrum of transformation and the outcomes are also heterogenous. Some exhibit aggressive clinical features and treatment resistance. Multiple therapies may be considered at an earlier time point and a long-period follow up is necessary. The mechanisms of transformation are still unknown. The existing hypothesis seem hard to explain the transformation with the heterogeneous spectrum completely. PC1/3, HWM ACTH and gene mutation may be involved in the mechanisms underlying transformation. Future studies using transcription factor detection and genome sequencing may be useful to explore the underlying mechanism of this rare transformation.

The major limitation of this review is the case size is small. On the one hand, this phenomenon is indeed rare; on the other hand, the non-uniform definition and name may have led us to miss some of the literature. Thus, clinicians need to recognize this rare phenomenon more clearly, and more potential cases are needed to clarify the features and guide clinical decisions. These particular PitNETs are precious material for figuring out the mechanism of secretary regulation and differentiation in PitNETs [45]. Like PC1/3 and erythropoietin-producing hepatocellular receptor 6, we can find or validate more regulation mechanisms through multi-omics analysis, for the tumors have minimal individual differences between the two phases [45,64]. However, in addition to the rarity of it, there is no way to predict the biological behavior so that surgeons can preserve specimens purposefully. A precise prediction model may help solve this problem [65].
